# Browning of adipose tissue and increased thermogenesis induced by Methotrexate

**DOI:** 10.1096/fba.2021-00058

**Published:** 2021-10-07

**Authors:** Narendra Verma, Luce Perie, Carmen Corciulo, Philipp Leucht, Bhama Ramkhelawon, Bruce N. Cronstein, Elisabetta Mueller

**Affiliations:** ^1^ Division of Endocrinology, Diabetes and Metabolism Department of Medicine New York University Grossman School of Medicine New York NY USA; ^2^ Division of Translational Medicine Department of Medicine New York University Grossman School of Medicine New York NY USA; ^3^ Department of Orthopedic Surgery NYU Grossman School of Medicine New York NY USA; ^4^ Division of Vascular and Endovascular Surgery Department of Surgery New York University Grossman School of Medicine New York NY USA

**Keywords:** brown and beige fat, MTX, obesity, thermogenesis, UCP1

## Abstract

Methotrexate (MTX) is widely used for the treatment of rheumatoid arthritis due to its well‐known anti‐inflammatory role in immune cells but its impact on brown and beige adipose tissue biology has not yet been investigated. Here, we present the novel evidence that MTX treatment increases the gene expression of thermogenic genes in brown and beige adipose tissues in a fat cell autonomous manner. Furthermore, we show that treatment of mice with MTX is associated with cold resistance, improved glucose homeostasis, decreased inflammation, and reduced hepatosteatosis in high‐fat diet states. Overall, our data provide novel evidence of a role of MTX on thermogenic tissues not previously appreciated.

**Abbreviations**
ALT: alanine aminotransferase
AMPK: AMP‐Activated Protein Kinase
AST: Aspartate aminotransferase
ATGL: Adipose triglyceride lipase
BAT: Brown adipose tissue
eWAT: Epididymal adipose tissue
GTT: Glucose tolerance test
HFD: High‐fat diet
HSF1: Heat shock protein 1
IL6: Interleukin 6
ITT: Insulin tolerance test
MCP1: Monocyte chemoattractant protein‐1
MMP 14: Matrix Metallopeptidase 14
MTX: Methotrexate
PGC‐1α: Peroxisome proliferator‐activated receptor gamma coactivator 1‐alpha
PRDM16: PR domain containing 16
RA: rheumatoid arthritis
scWAT: Subcutaneous adipose tissue
TIMP 2: Tissue Inhibitor of Metalloproteinases 2
TNF‐α: Tumor necrosis factor‐alpha
UCP1: Uncoupling protein 1
ZFP516: Zinc finger protein 516
ZNF638: Zinc finger protein 638

## 1 INTRODUCTION

Brown and beige adipose tissues represent critical endocrine organs that regulate glucose and lipid homeostasis. It is now recognized that these tissues are present also in humans beyond the perinatal stage, persist throughout adulthood, and decline during aging. We and others have demonstrated that brown/beige adipocytes are critically involved in the control of metabolism through their effects on glucose homeostasis^1^, ^2^, ^3^, ^4^ and shown that their activation can improve diabetes.^1^

One of the key genes involved in beige and brown adipose tissue functionality is the uncoupling protein 1 (UCP1), a protein that uncouples respiration from energy production leading to heat generation.^5^ In addition to UCP1, it has been shown that factors such as CIDEA, PGC‐1α and DIO2 are critically involved in brown and beige fat functionality and are highly induced during browning in subcutaneous white adipose tissue (scWAT) upon cold stimuli or pharmacological activation of β‐adrenergic signaling pathways.^6^, ^7^

Methotrexate (MTX) is a chemotherapy agent and immune‐system suppressant initially used for the treatment of malignancies such as lymphoma, acute lymphoblastic leukemia, and osteosarcoma.^8^, ^9^, ^10^, ^11^, ^12^, ^13^, ^14^ In the last several decades, MTX has been successfully employed for the treatment of rheumatoid arthritis (RA)^9^, ^14^ due to its potent anti‐inflammatory activity, and is now the standard mode of treatment for this disease. In a recent trial, involving nearly 5000 patients taking either low‐dose MTX or placebo for the prevention of atherosclerotic events, there was a significant increase in individuals experiencing weight loss in the MTX cohort compared to the placebo group.^15^

Here we provide novel evidence demonstrating that mice treated with MTX have decreased weight, increased adipose tissue browning, and enhanced thermogenesis. Our in vitro data obtained in isolated cells reveal that MTX activates beige/brown fat gene expression programs in a cell autonomous manner. Furthermore, we demonstrate that MTX treatment of mice on a high‐fat diet (HFD) is associated with decreased body weight, improved insulin resistance, and reduced inflammation in fat and decreased hepatosteatosis and hepatosteatitis. Overall, our studies demonstrate for the first time that MTX affects brown/beige adipose tissue biology and provide new insights into the metabolic function of MTX.

## 2 MATERIALS AND METHODS

### 2.1 Cell culture

10T1/2 cells were obtained from the American Type Culture Collection (ATCC). For stromal vascular craction (SVF) cells isolation, subcutaneous/inguinal fat pads (scWAT) were obtained from male mice after they were euthanized by cervical dislocation, according to procedures previously described.^16^ Specifically, scWAT depots were dissected from 9‐week‐old C57BL/6J male mice, washed in 1× PBS buffer (Corning, #21‐040‐CV) supplemented with 1% pen/strep, minced into small pieces, and digested with 1 mg/ml type IV collagenase (Roche, #10269638001) at 37℃ for 1 h, while shaking. The cell suspension obtained was then filtered through a 70 µm cell strainer (BD Falcon, #352350) to eliminate clumps of undigested cells and subsequently span at 200 × *g* for 10 min. The cells of the SVF present in the pellet were re‐suspended in medium containing DMEM (Corning, #10‐013‐CV), supplemented with 10% FBS, and 1% pen/strep, and plated in 12‐well tissue culture plates, according to procedures previously described.^17^ 10T1/2 cells and SVF cells were maintained in culture medium (DMEM) supplemented with 10% of fetal bovine serum (FBS, Thermo Fisher Scientific, # NC0959573) and 1% penicillin/streptomycin (pen/strep, Thermo Fisher Scientific, #15070063). Brown‐like adipocyte differentiation was induced by treating confluent 10T1/2 cells, or SVF cells, with DMEM medium containing 10% FBS, and 1% pen/strep, supplemented with 20 nM insulin (Sigma, #I1507), 1 nM T3 (Sigma, #T2877), 125 µM indomethacin (Sigma, #I7378), 1 µM dexamethasone (Sigma, #D4902), 1 µM rosiglitazone (Sigma, #557366‐M), and 0.5 µM isobutylmethylxanthine (IBMX; Sigma, #I5879). After 48 h of stimulation for 10T1/2 cells, or after 120 h for SVF cells, the induction medium was removed and a maintenance medium, containing DMEM supplemented with 10% FBS, 1% pen/strep, 1 nM T3, and 20 nM insulin, was added and replaced every 2 days. To induce white fat differentiation, 10T1/2 cells were induced with a differentiation medium containing 0.5 μM isobutylmethylxanthine, 1 μM dexamethasone, and 5 μg/ml insulin, and subsequently maintained in medium containing DMEM medium supplemented with 10% FBS, 1% pen/strep, and 20 nM insulin and replaced every 2 days. Once differentiated, adipocytes were treated with either vehicle (DMSO) or MTX (Teva Pharmaceuticals, NDC# 00703‐3671‐01), for 24 h at the concentrations indicated in the legends, prior to harvesting the cells for RNA or protein analysis.

### 2.2 Mice

Mice were exposed to a 12‐h light/12‐h dark cycle with free access to food and water. For in vivo MTX treatment, C57BL/6J 8‐week‐old male mice were purchased from Jackson Laboratories (cat #000664), housed in the animal facility at 24℃ for 1 week for their acclimation, before they were used for any of the experiments described. To study the long‐term effects of pharmacological stimulation of MTX, 9‐week‐old male C57BL/6J mice were placed on HFD diet (60% Kcal fat, Research Diet, D124920) and injected i.p. once a week for 20 weeks with either saline or with MTX (Teva Pharmaceuticals, NDC# 00703‐3671‐01) dissolved in saline at the concentration of 1 mg/kg/body weight. For cold tolerance tests, 9‐week‐old WT mice maintained on a chow diet were injected i.p twice a week for 4 weeks with 1 mg/kg of MTX, or vehicle, and subsequently placed at 4℃ for up to 6 h. Measurements of core temperature were obtained from mice individually caged and exposed to 4℃ with free access to water. Core body temperature was monitored using a rectal thermometer (BAT‐12, Physitemp) before the start of cold exposure, and every hour for 6 h. At the end of the cold exposure period, mice were euthanized and fat depots were harvested for analysis. All animal procedures used in this study were approved by the Institutional Animal Care and Use Committee of NYU Langone Medical Center.

### 2.3 RNA isolation and RT‐PCR analysis

Total RNA was extracted from cells using RNeasy (Qiagen, #75144) and from tissues using TRIzol (Thermo Fisher Scientific, #15596018). One microgram of total RNA was reverse transcribed into cDNA using the iScript™ cDNA Synthesis Kit (Bio‐Rad, #1708890). RT‐PCR analysis was performed in triplicate, using 25 ng of cDNA, 300 nM of primers (see below), and iQ™ SYBR® Green Supermix (Bio‐Rad, #1708880), following the manufacturer's instructions. The ΔΔCt method was used for relative mRNA quantification by normalizing each sample to the average change in cycle threshold value of the 36B4 gene used as a control. The following primers were used for Q‐PCR analysis: UCP1 Fwd: GGCCCTTGTAAACAACAAAATAC UCP1 Rev: GGCAACAAGAGCTGACAGTAAAT; DIO2 Fwd: AATTATGCCTCGGAGAAGACCG; DIO2 Rev: GGCAGTTGCCTAGTGAAAGGT; CIDEA Fwd: TGACATTCATGGGATTGCAGAC; CIDEA Rev: CGAGCTGGATGTATGAGGGG; PGC‐1α Fwd: ACCATGACTACTGTCAGTCACTC; PGC‐1α Rev: GTCACAGGAGGCATCTTTGAAG; ATGL Fwd: TGTGGCCTCATTCCTCCTAC; ATGL Rev: TCGTGGATGTTGGTGGAGCT; Cd36 Fwd: TTTGGAGTGGTAGTAAAAAGGGC; Cd36 Rev: TGACATCAGGGACTCAGAGTAG, aP2 Fwd: ACACCGAGATTTCCTTCAAACTG; aP2 Rev: CCATCTAGGGTTATGATGCTCTTC IL‐6 Fwd: GACAACTTTGGCATTGTGG; IL‐6 Rev: ATGCAGGGATGATGTTCTG; TNF‐α Fwd: CCAGACCCTCACACTCAGATC; TNF‐α Rev: CACTTGGTGTGCTACGAC; MCP1 Fwd: AGGTCCCTGTCATGCTTCTG; MCP‐1 Rev: GCTGCTGGTGATCCTCTTGT; ZNF638 Fwd: ATTGAGAGCTGTCGGCAGTTA; ZNF638 Rev: GGAATGAG AACGT CTTCTTGGAG; ZFP516 Fwd: AGCGCTTGGATATCCTCAGTA, ZFP516 Rev: GAGGGGCCCTGCTGGCACAGT; PRDM16 Fwd: CCACCAGCGAGGACTTCAC; PRDM16 Rev: GGAGGACTCTCGTAGCTCGAA; HSF1 Fwd: AGGCAGGAGCATAGATGAGA; HSF1 Rev: AGGATGGAGTCAATGAAGG; Cd68 Fwd: TGTCTGATCTTGCTAGGACCG; Cd68 Rev: TGTCTGATCTTGCTAGGACCG; Cd206 Fwd: CTCTGTTCAGCTATTGGACGC; Cd206 Rev: CGGAATTTCTGGGATTCAGCTTC; Ym1 Fwd: GTCTTGCTCATGTGTGTAAGTGA; Ym1 Rev: CAGGTCTGGCAATTCTTCTGAA. MMP14 Fwd: CCCTAGGCCTGGAACATTCT; MMP14 Rev: TTTGGGCTTATCTGGGACAG TIMP2 Fwd: ACAGGCGTTTTGCAATGCA; TIMP2 Rev: GGGTTGCCATAAATGTCGTTTC.

### 2.4 Western Blot analysis and antibodies

Protein extracts were obtained after differentiated 10T1/2 or SVF cells were washed for at least three times with cold PBS. Snap frozen tissues were homogenized with a tissue grinder (Powergen 700, Thermo Fisher Scientific, Cat # GLH 115) and lysed in RIPA buffer, containing 20 mM Tris, 150 mM NaCl, 1% Triton X‐100, and supplemented with a cocktail of protease inhibitors (Thermo Fisher Scientific, #PIA32953). Twenty micrograms of protein lysates was subsequently run on 10% SDS‐polyacrylamide gels and transferred on a 0.45 μm PVDF membrane (Millipore, #IPVH00010). Blots were subsequently pre‐incubated at room temperature (RT) for 1 h, with 5% nonfat dry milk (w/v) resuspended in 0.1% TBST buffer, containing 50 mM Tris‐HCl, 150 mM NaCl, pH 7.4, and 0.1% Tween‐20, and subsequently incubated in a 0.1% TBST buffer solution containing primary antibodies (see below), overnight at 4℃ (Thermo Fisher Scientific; BP9703‐100). The following antibodies, at the indicated dilutions, were used for the analyses: anti‐UCP1 (Abcam, #ab23841) at 1:1000, anti‐Phospho‐AKT Ser473 (CST, #9271) at 1: 1000, AKT (CST, #9272) at 1:1000, anti‐AMPK (CST, # 2532) and anti‐vinculin (Proteintech, #66305‐1‐Ig) at 1:2000. After primary antibody incubation, membranes were washed in TBST 0.1% (v/v) and incubated for 1 h at RT with an anti‐rabbit (Bio‐Rad; #1706515) or anti‐mouse (Bio‐Rad; #1706516) IgG horseradish peroxidase‐conjugated antibody, in 0.1% TBST containing 2% nonfat dry milk (w/v) at a dilution of 1:20,000. After four additional washes in TBST 0.1% (v/v), blots were developed using enhanced chemiluminescence (GE Healthcare, #RPN2108), using Hyblot CL autoradiography films (Thomas Scientific #E3012), and developed with an X‐ray film developer (Konica Minolta, #SRX‐101A). ImageJ (Research Services Branch, National Institute of Mental Health, Bethesda, MD) was used for densitometric analysis of western blots. Each sample was normalized to the signal intensity of vinculin used as a loading control in each experiment. Control values were set to 1.0. For densitometry analysis, the quantitation was performed within the linear range of the standard curve defined by the standard sample or vinculin.

### 2.5 Histology

Dissected tissues were fixed in 10% neutral buffered formalin and embedded in paraffin, according to the standard procedures. Tissue sections of scWAT, BAT, and liver of 5‐mm thickness were stained with H&E, with an anti‐UCP1 antibody (scWAT and BAT) (Abcam, ab10983) or with an anti‐F4/80 antibody (liver) (Abcam, ab6640), following the manufacturer's instructions.

### 2.6 GTT and ITT

GTT and ITT were measured after 4–5 weeks of HFD. For GTT analysis, mice were fasted overnight and injected i.p. with glucose in saline solution (2 g/kg) and plasma glucose levels were measured at 0, 15, 30, 60, 90, and 120 min after glucose injections using tail blood. For ITT analysis, mice received an intra‐peritoneal injection of insulin in saline solution (1 mU/kg) and plasma glucose levels were measured at 0, 15, 30, 60, 90, and 120 min.

### 2.7 Serum parameters

We analyzed AST and ALT levels in sera obtained from mice treated either with vehicle or MTX (1 mg/kg), using colorimetric assays (Sigma, Cat# MAK055 and Cat# MAK052), according to the protocols provided by the vendors.

### 2.8 Statistical analysis

The results were expressed as a mean ± standard error (SE), unless otherwise noted. Student's *t*‐test or one‐way analysis of variance (ANOVA) was used for comparison between groups. *p* values < 0.05 were considered to be statistically significant. Statistical analyses were performed using the Prism 7 software (GraphPad Software).

## 3 RESULTS

### 3.1 MTX induces browning fat cell autonomously

**FIGURE 1:**
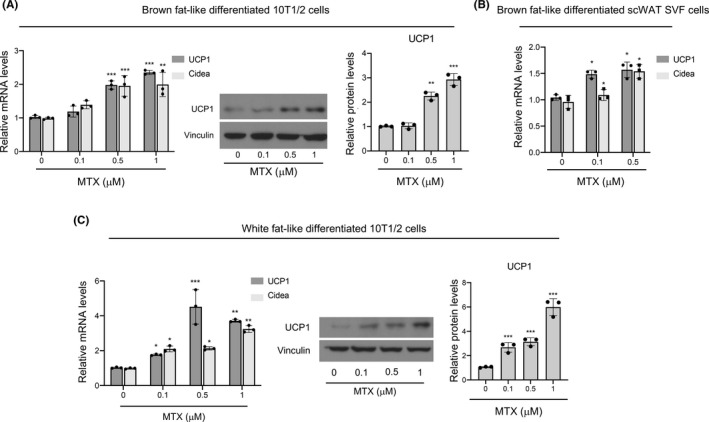
Methotrexate induces browning in a fat cell autonomous manner. (A) UCP1 and CIDEA relative mRNA levels and protein levels of UCP1 are presented as western blot and quantified via densitometric analysis in brown differentiated 10T1/2 cells treated with vehicle (0) or with 0.1, 0.5, and 1 μM of MTX. Vinculin was used as a loading control. (B) Relative mRNA levels of UCP1 and CIDEA in brown differentiated SVF cells obtained from scWAT of WT mice treated with vehicle or MTX. (C) UCP1 and CIDEA relative mRNA and protein levels of UCP1 are presented as western blot and quantified via densitometric analysis in white differentiated 10T1/2 cells treated with vehicle (0) or with 0.1, 0.5, and 1 μM of MTX. Vinculin was used as a loading control. Results are expressed as a mean ± SEM from three independent experiments and *p < 0.05; **p < 0.005; ***p < 0.001

Given that MTX treatment in human has been associated with changes in body weight^15^, we sought to determine whether MTX could affect adipocyte biology and possibly regulate thermogenic gene expression and browning. To assess this, we measured the levels of brown fat markers in white and brown‐like differentiated 10T1/2 cells exposed to different doses of MTX. MTX treatment was associated with significant increase in the mRNA levels of UCP1 and CIDEA in differentiated 10T1/2 cells (Figure 1A) and in SVF cells obtained from scWAT (Figure 1B) and did not appear to affect the cell viability, given the absence of floating cells in the culture medium. Similar effects on gene expression were also observed when 10T1/2 cells differentiated into white fat (Figure 1C) were treated with MTX. Furthermore, the effects of MTX on thermogenic gene expression were accompanied by changes in UCP1 protein levels (Figure 1A and C). These results demonstrate that MTX treatment can induce brown fat markers in isolated adipocytes.

### 3.2 MTX treatment increases cold tolerance

**FIGURE 2:**
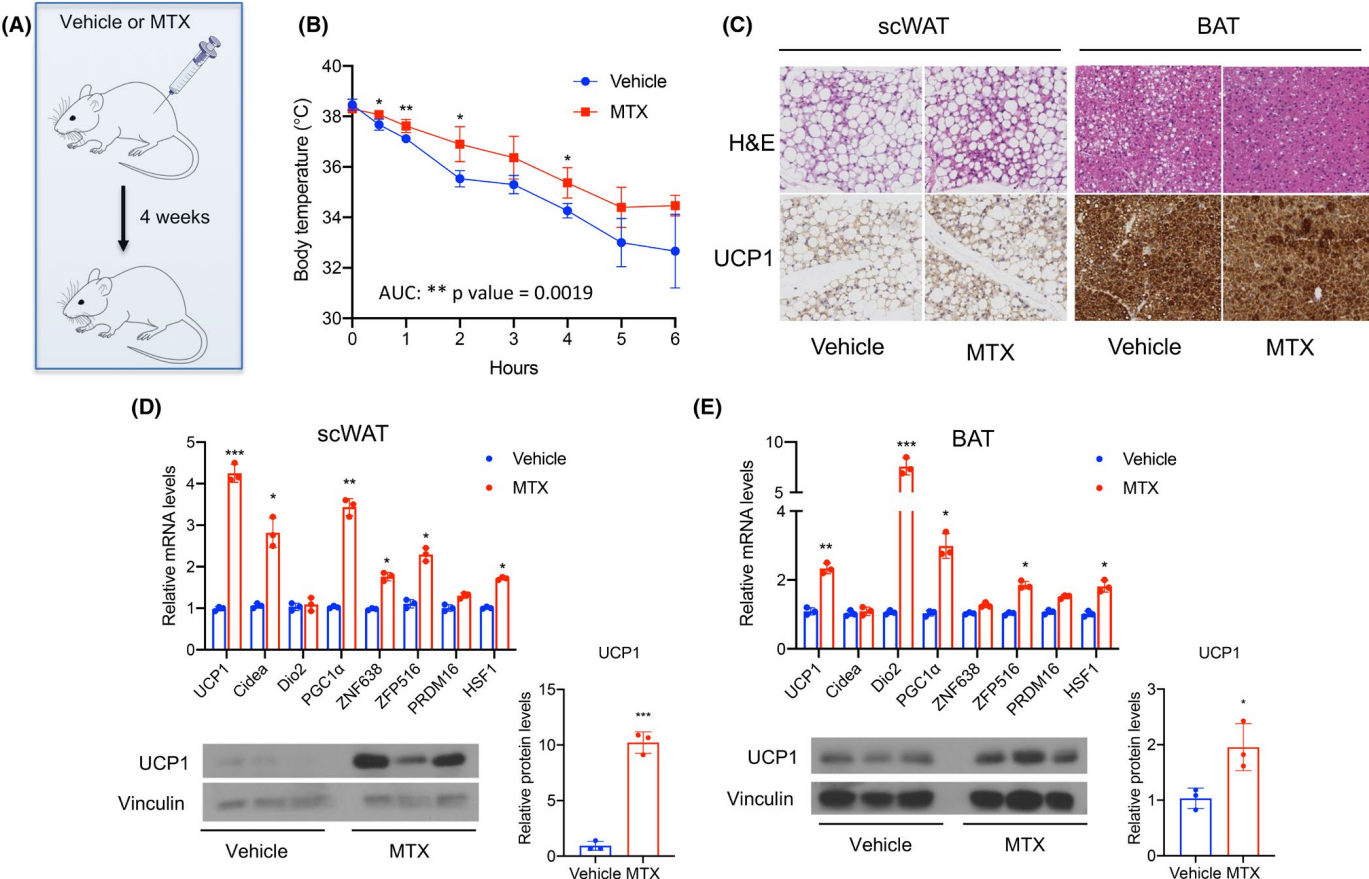
MTX increases adaptive thermogenesis. (A) Schematic representation of the experimental design, (B) core temperatures during cold exposure, (C) H&E and UCP1 staining of scWAT and BAT, and relative mRNA levels of UCP1, DIO2, CIDEA, PGC‐1α, ZNF638, ZFP516, PRDM16, and HSF1 and protein levels of UCP1 are presented as western blot and quantified via densitometric analysis in scWAT (D) and BAT of mice (E) on standard chow diet treated twice a week with saline (vehicle) or with 1 mg/kg MTX for 4 weeks. Magnification, ×40; scale bar  = 100 μm. Experiments were performed in C57BL/6J male mice, three mice per group. Results are expressed as a mean ± SEM and *p < 0.05; **p < 0.005; ***p < 0.001

Given that MTX induces UCP1 in vitro, we tested whether MTX could modulate thermogenic responses through induction of browning in vivo. We, therefore, treated C57BL/6J male mice i.p at 9 weeks of age, twice a week, with either vehicle or MTX (1 mg/kg) for 4 weeks and subsequently exposed them to cold temperatures (4℃) for 6 h (Figure 2A). We chose the 1 mg/kg dose of MTX to exclude potential effects of MTX on feeding, given that MTX at comparable doses was shown to have no impact on food intake.^18^ Mice receiving MTX showed increased cold tolerance, compared to control mice (Figure 2B). The MTX‐treated mice also showed a reduction in body weight compared to control‐treated mice, which was associated with a trend in decrease of fat tissue amounts (Figure S1). To determine whether the increased thermogenic resistance of MTX‐treated mice was associated with enhanced fat tissue browning, we performed histology and molecular analysis of adipose depots obtained from MTX‐ and control‐treated mice. H&E staining and immunohistochemistry revealed decreased lipid accumulation and increased UCP1 staining in scWAT and BAT of MTX‐treated mice, compared to control mice (Figure 2C). Analysis of mRNA levels of brown fat markers in adipose tissues confirmed that exposure to MTX was associated with increased levels of UCP1, CIDEA, and DIO2 and of genes such as ZNF638, ZFP516, PRDM16, and HSF1 (Figure 2D and 2E) known to be induced in response to cold exposure in thermogenic tissues.^16^, ^17^, ^19^, ^20^, ^21^ These browning effects were also accompanied by increased UCP1 protein levels in both scWAT and BAT (Figure 2D and 2E). All together, these data demonstrate that MTX‐mediated browning of fat tissues in vivo is associated with cold resistance.

### 3.3 Improved glucose homeostasis in methotrexate‐treated mice

**FIGURE 3:**
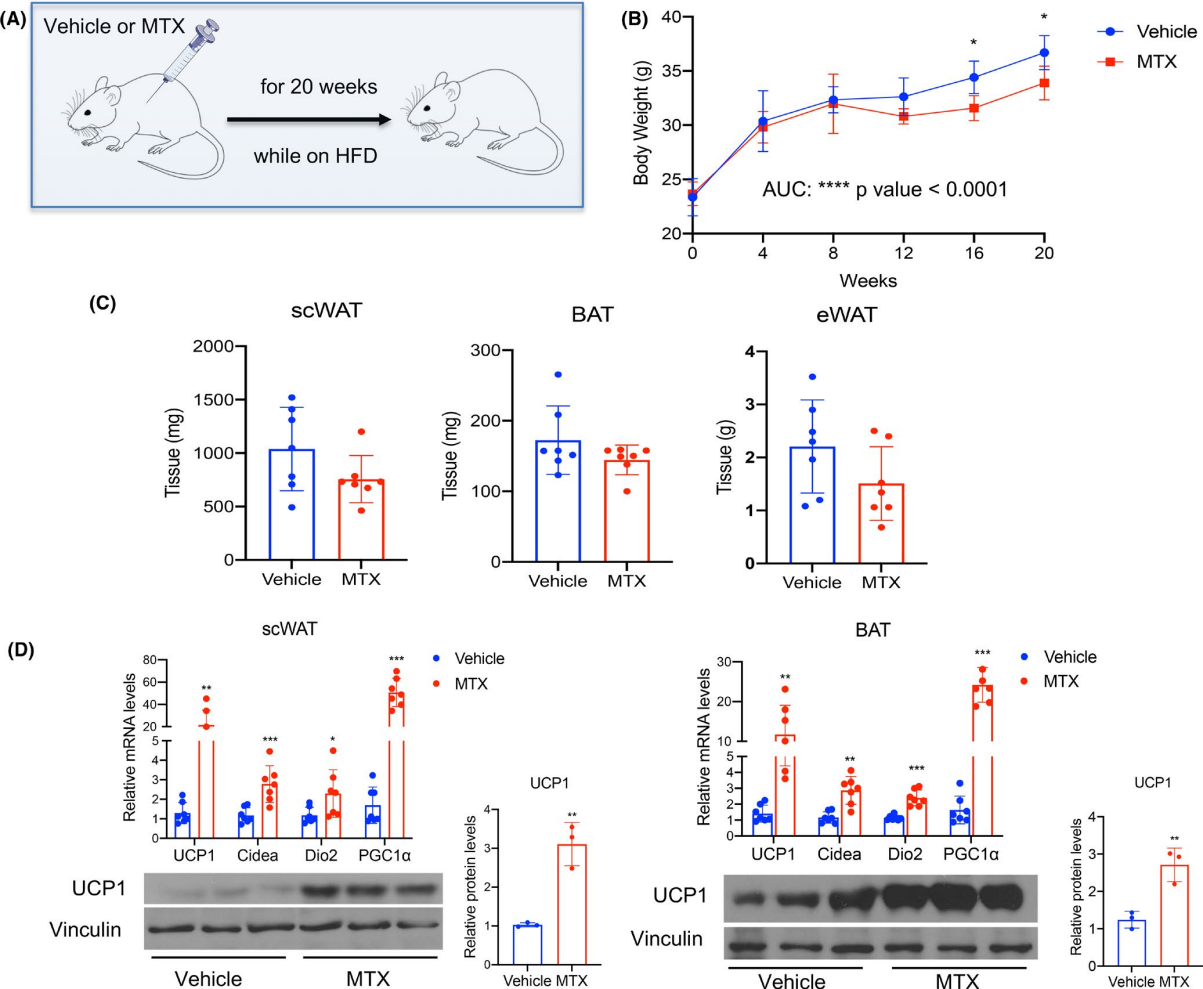
MTX treatment of mice on HFD is associated with adipose tissue browning. (A) Schematic representation of the experimental design, (B) body weight, (C) adipose tissue weight, and (D) relative mRNA levels of UCP1, DIO2, CIDEA, and PGC‐1α and protein levels of UCP1 are presented as western blot and quantified via densitometric analysis in scWAT and BAT of mice on HFD treated once a week with either saline (vehicle) or with 1 mg/kg MTX for 20 weeks. Experiments were performed in C57BL/6J male mice, *n* = 7 per group. Results are expressed as a mean ± SEM and *p < 0.05; **p < 0.005; ***p < 0.001

**FIGURE 4:**
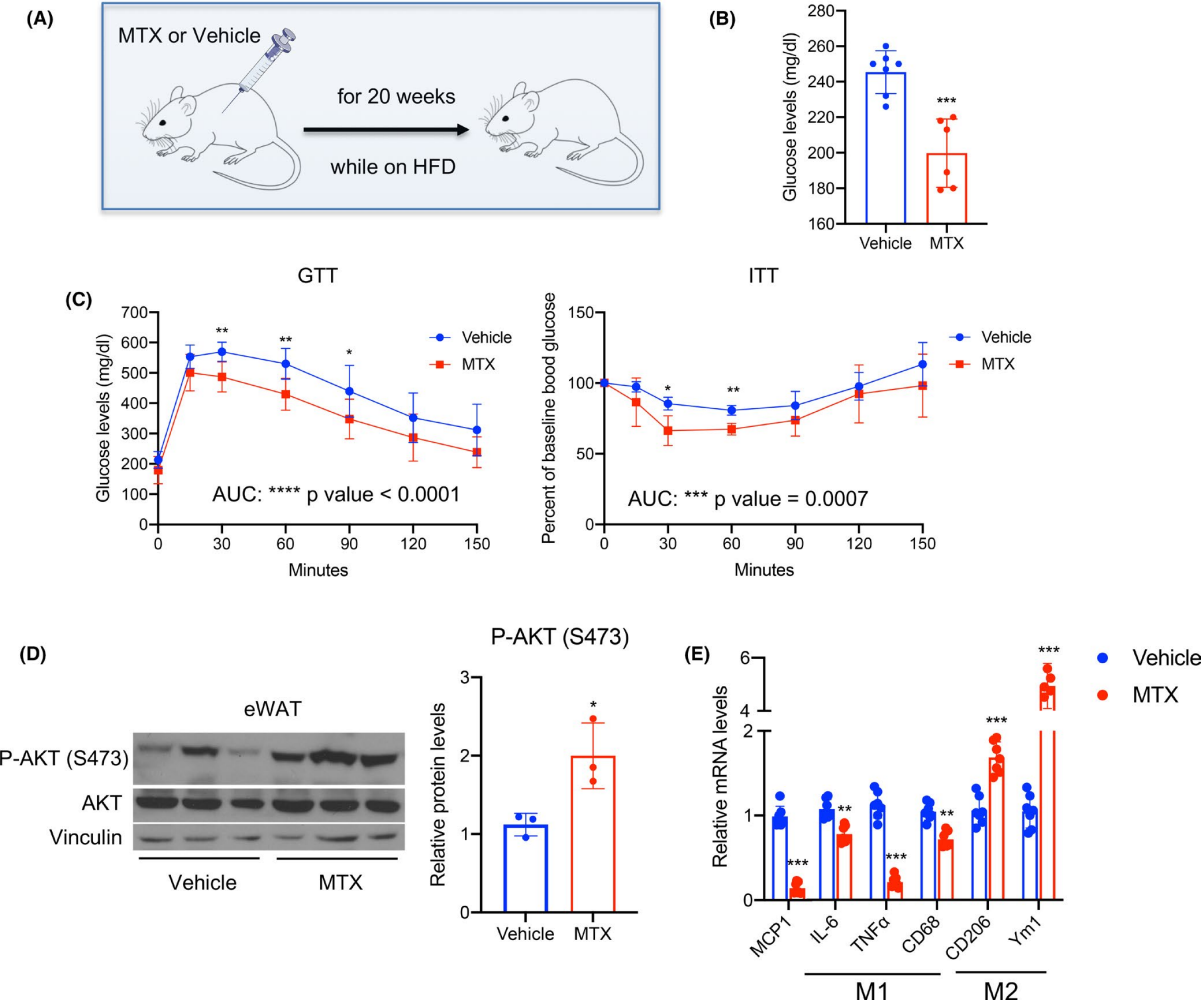
MTX treatment of DIO mice is associated with improved insulin sensitivity. (A–E) Analysis of mice on HFD, treated with saline (vehicle) or with 1 mg/kg MTX, for 20 weeks, once a week. (A) Schematic representation of the experimental design, (B) glucose levels after 4 h fasting, (C) GTT and ITT, (D) protein levels of phospho‐AKT (S473) and total AKT levels are presented as western blot and quantified via densitometric analysis in eWAT, and (E) relative mRNA levels of pro‐inflammatory genes and M1 and M2 macrophages markers in eWAT. Experiments were performed in C57BL/6J male mice, *n* = 7 per group. Results are expressed as a mean ± SEM and *p < 0.05; **p < 0.005; ***p < 0.001

To assess the effects of MTX on metabolic dysfunction driven by nutritional overload, we treated 9‐week‐old C57BL/6J male mice i.p with saline (vehicle) or with MTX (1 mg/kg) for 20 weeks during exposure to HFD (Figure 3A). As shown in Figure 3B and Figure S2, MTX treatment was associated with decreased body weight in mice on HFD compared to control‐treated mice. To exclude that the changes observed were due to toxic effects of MTX, we measured ALT and AST in the sera of control‐ and MTX‐treated mice and demonstrated no differences in the levels of these liver enzymes between the two groups (Figure S3). Analysis of tissue amounts in control‐ and MTX‐treated mice revealed no differences in adipose tissue weights, suggesting that fat tissues do not account for the difference in body weight observed (Figure 3C). Furthermore, molecular analysis of scWAT and BAT revealed increased expression of brown fat markers (Figure 3D). Given the evidence that we and others have provided that activation of brown fat function is associated with improved glucose homeostasis,^1^, ^22^ we assessed whether MTX treatment was accompanied by changes in insulin sensitivity (Figure 4A). Our analysis revealed that mice on HFD treated with MTX are more insulin sensitive, as shown by the decreased blood glucose levels in MTX‐treated mice compared to control‐treated mice (Figure 4B) and by GTT and ITT assays (Figure 4C). Moreover, we found that phospho‐AKT (p‐AKT‐S473) levels were significantly increased both in eWAT and in liver (Figure 4D and Figure S4), further supporting the notion that MTX improves insulin sensitivity, while no differences in the levels of AMPK, in muscle of control‐ and MTX‐treated mice were observed (Supplementary Figure S4). To determine whether MTX treatment was associated with changes in inflammation in eWAT, we analyzed a panel of genes encoding for proteins involved in this process. Our data demonstrate that the eWAT of MTX‐treated mice has reduced expression of pro‐inflammatory genes, such as MCP1 and IL‐6, TNF‐α and CD68, and increased levels of CD206 and Ym1, two markers of M2 macrophages. These results reveal that MTX treatment is associated with alleviation of insulin resistance, reduced inflammation, and increased M2 polarization in white adipose tissue of mice on HFD (Figure 4E).

### 3.4 Low‐dose MTX treatment is associated with protection from fatty liver and steatitis

**FIGURE 5:**
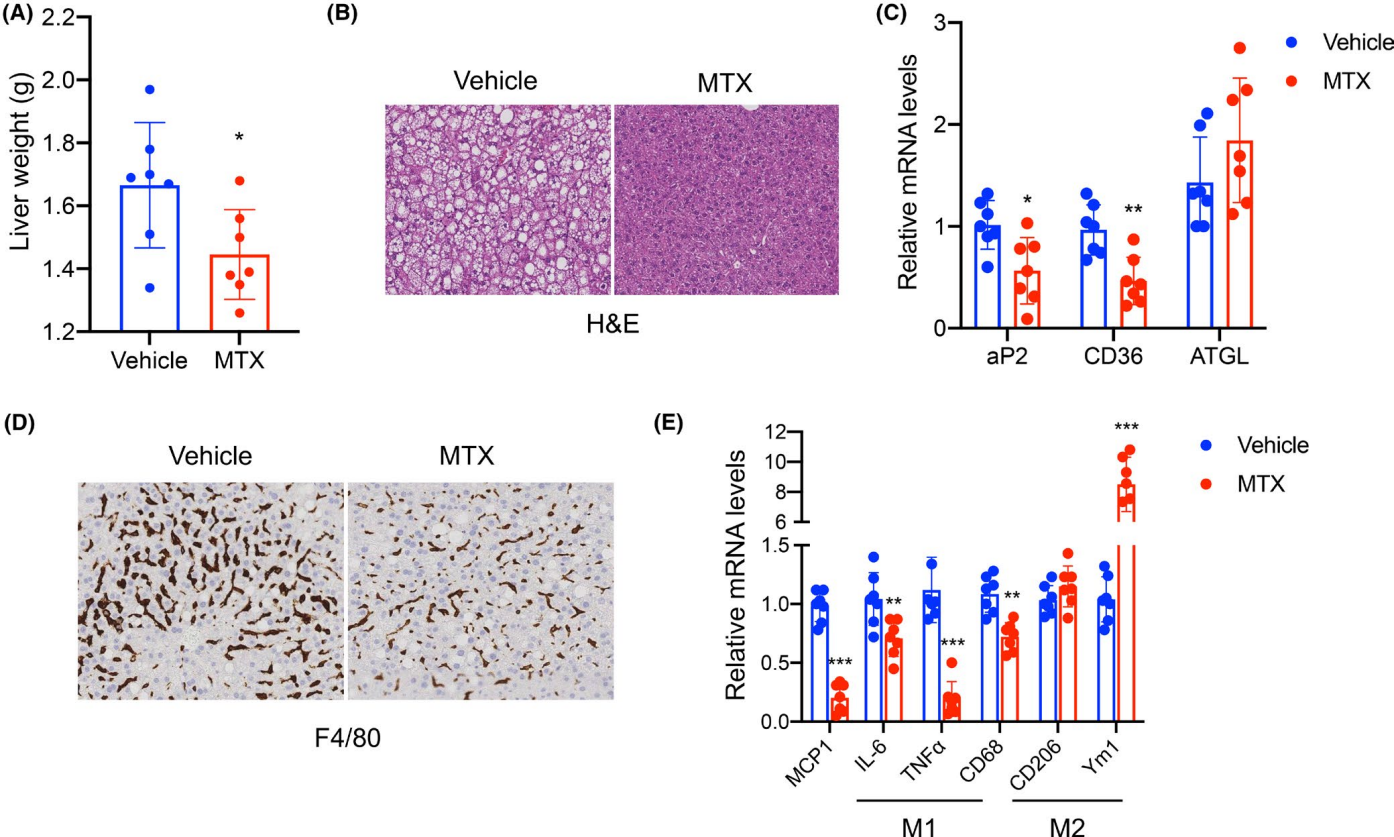
MTX treatment is associated with protection from fatty liver and hepatosteatitis. (A) Liver tissue weight, (B) H&E staining, (C) relative mRNA levels of aP2, CD36, and ATGL, (D) F4/80 staining, and (E) relative mRNA levels of pro‐inflammatory genes and M1 and M2 macrophages markers in liver of mice on HFD treated with saline (vehicle) or 1 mg/kg MTX for 20 weeks, once a week. Magnification, ×40; scale bar = 100 μm. Experiments were performed with C57BL/6J male mice, *n* = 7 per group. Results are expressed as a mean ± SEM and *p < 0.05; **p < 0.005; ***p < 0.001

**FIGURE 6:**
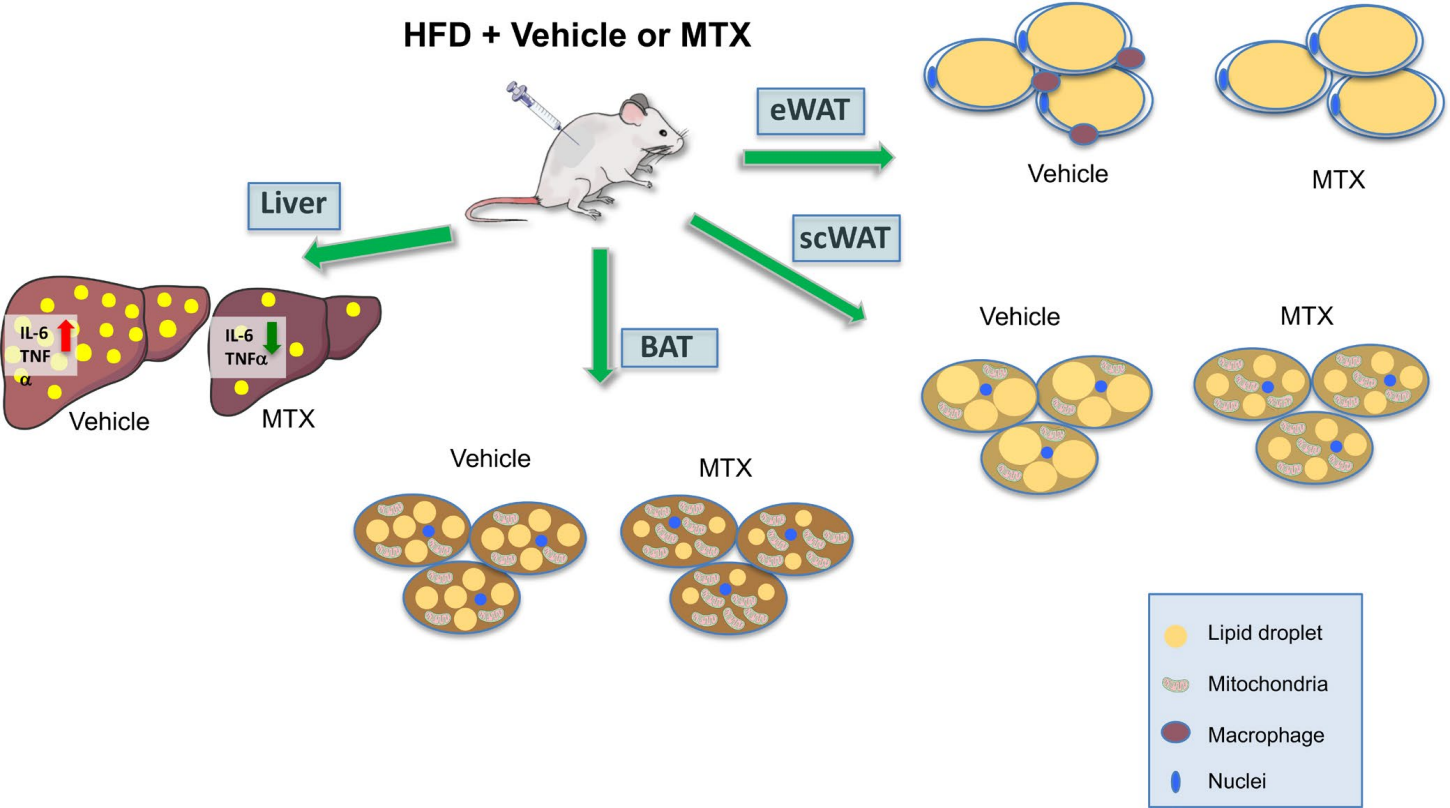
Schematic representation of the effects of low doses of MTX on thermogenic fat and liver. MTX treatment is accompanied by browning of fat tissue and decreased inflammation, and is associated with protection from hepatic steatosis and steatitis

Obesity is often accompanied by ectopic lipid accumulation leading to hepatosteatosis and, eventually, hepatosteatitis. Our analysis of mice exposed to HFD showed a significant reduction specifically in liver weight of MTX‐treated mice compared to controls (Figure 5A), while other tissues showed no significant differences (Figure S5). To assess the amount of ectopic fat deposition present in livers of control‐ and MTX‐treated mice, we performed both histological and molecular analyses of liver biopsies obtained from these mice. H&E staining indicated reduced lipid accumulation in mice receiving MTX (Figure 5B) and RNA analysis demonstrated a decrease in the expression of genes contributing to lipid deposition, such as aP2 and CD36, and a trend in increase in the levels of the lipolytic gene ATGL (Figure 5C) in these mice. Furthermore, histological analysis of macrophage infiltration revealed reduced F4/80 staining in liver biopsies of MTX‐treated mice (Figure 5D). To further quantify the effects of MTX on inflammation, we measured the levels of a number of markers involved in this process. Our results demonstrate reduced expression of pro‐inflammatory genes and markers of M1 and an increase in M2 markers in livers of MTX‐treated mice, compared to controls (Figure 5E). To further exclude any possible toxic effects of MTX on liver, we measured the levels of two known markers of fibrosis, MMP 14 and TIMP 2. This analysis revealed no differences in the expression of these genes in the livers of control‐ and MTX‐treated mice (Suppl. Figure S6) suggesting the absence of adverse side effects of MTX treatment. Altogether, our data demonstrate that MTX improves thermogenic function and metabolism in obese states (Figure 6).

## 4 DISCUSSION

In the last three decades, MTX has become the standard of care for the treatment of rheumatoid arthritis.^23^ Despite the wealth of knowledge on the anti‐inflammatory action of MTX accumulated over the years, the potential effects of MTX on beige and brown adipose tissue biology are currently unknown. Here, we show for the first time that MTX treatment in mice leads to increased thermogenic responses and is accompanied by the induction of brown fat gene expression. In conditions of nutrition overload, browning induced by low doses of MTX treatment is associated with protection from the development of insulin resistance, from hepatosteatosis, and from inflammation in adipose tissues and liver.

MTX has been previously shown to have anti‐inflammatory effects on adipose tissue^18^ and to improve glucose metabolism in muscle,^24^ but whether MTX affects thermogenesis has not yet been tested. We hypothesized that low doses of MTX may induce browning in adipose tissues and increase thermogenic responses, triggering beneficial metabolic changes, without any of the negative side effects on other organs previously reported.^25^ Our in vivo data indicating improved cold tolerance in short‐term MTX‐treated mice via browning of adipose tissue was supported by in vitro studies performed in isolated, differentiated adipocytes, demonstrating that MTX increases brown fat gene expression in a cell autonomous manner. Altogether our data are the first to suggest a link between MTX and thermogenesis.

We demonstrate that livers of MTX‐treated mice have decreased lipid accumulation and inflammation after HFD, compared to control mice. These results suggest for the first time that MTX may improve the hepatosteatosis and hepatosteatitis conditions that usually occur in response to nutritional overload. Our data on the effects of MTX on the liver are different from those obtained from other published studies which have reported liver toxicity as a result of treatment with MTX.^25^, ^26^ It is plausible that the absence of MTX‐mediated hepatotoxicity in our study may be due to the low doses of MTX we have used. All together our data support the evidence that low doses of MTX can decrease hepatic steatosis and steatitis associated with nutrition overload.

It was recently shown that more patients taking MTX than placebo participating in a trial of MTX for the prevention of atherosclerotic events experienced an unintended weight loss. Most of the patients in this trial were suffering from diabetes and were overweight.^15^ Although there are many potential reasons for the unintended weight loss observed in the MTX patients in the aforementioned trial, it is plausible that induction of browning could account for the weight loss observed in these patients.

Our study shows that MTX treatment is associated with changes in overall body weight. While we observed a non‐statistically significant reduction of fat tissue amounts, the decrease in liver weight appears to be the major contributor to the reduction in total body weight. This evidence is supported by the decreased hepatic lipid storage detected by both histological and molecular analyses. Previously published data by Meyers and colleagues have shown decreased body weight and fatty liver as a result of MTX treatment of C57BL/6J, but the mechanisms had been attributed to a possible role of MTX on depletion of adipocyte precursors and on the suppression of hepatic lipogenesis. The effects on fat and liver reported in the Meyers study were observed after treatment with weekly doses of MTX that were 250 times higher than those we used in this study. In contrast, DeOliviera and colleagues, analyzed the effects of treatment with 4 mg/kg of MTX on Swiss mice on HFD and did not observe any change in body weight nor reported any browning in adipose tissue.^18^ It is possible that the differences between the effects of MTX observed in these studies and ours may be due to the different doses of MTX used and the mouse strains analyzed. It is plausible that low doses of MTX may be sufficient to induce browning and decrease ectopic fat accumulation in a strain‐dependent manner, given the known intrinsic differential propensity to develop diabetes or obesity in distinct mouse models.^27^

In conclusion, our data demonstrate that the use of MTX at low doses affects brown fat biology and alleviates the metabolic dysfunction that normally occurs in condition of prolonged nutritional overload.

## 5 DISCLOSURE SUMMARY

B.N.C. owns stocks in Regenosine. All the other authors have nothing to disclose.

## CONFLICT OF INTEREST

The authors declare that they have no conflict of interest with the contents of this article.

## AUTHOR CONTRIBUTIONS

NV, LP, CC, and EM designed the research; NV, LP, and CC performed the experiments; NV, LP, CC, PL, BR, BNC, and EM analyzed the data; CC, PL, BR, and BNC edited the manuscript; and NV, LP, and EM wrote the manuscript.

## Supporting information

Fig S1‐S6
