# Supplementary material for: Browning of adipose tissue and increased thermogenesis induced by Methotrexate
Source: FASEB Bioadv. 2021 Oct 7;3(11):877–87. doi: 10.1096/fba.2021-00058 (PMC8565234; doi:10.1096/fba.2021-00058)
Supplement: Supplementary file 1 — Fig S1‐S6 [file FBA2-3-877-s001.docx]

**
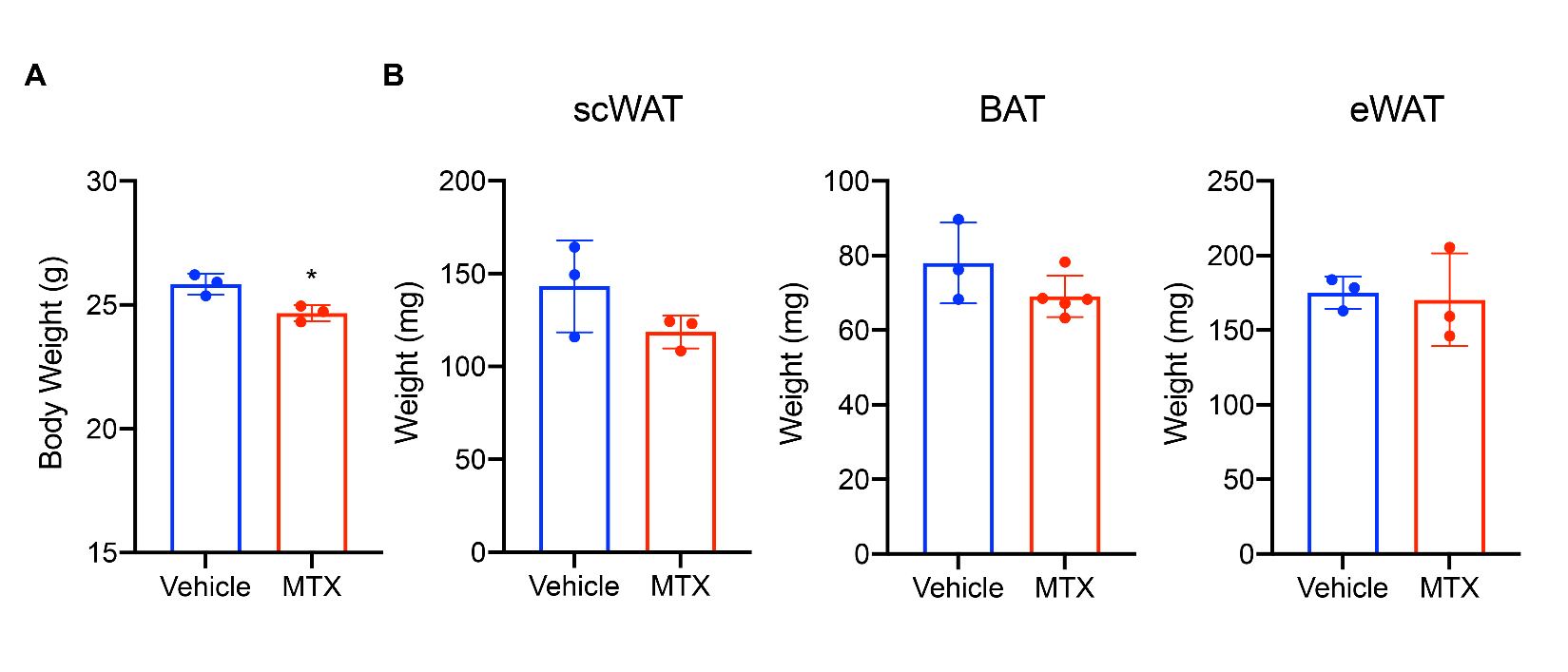
**

**Supplemental Figure 1.** **Body and fat tissue weight of mice treated with saline or MTX, twice a week, for 4 weeks, on standard chow diet.**

(**A**) Body weight and (**B**) fat tissue weight of mice on chow diet treated with vehicle or with 1mg/kg MTX for 4 weeks, twice a week. Experiments were performed with C57BL/6J male mice, n=3 per group. Results are expressed as a mean ± SEM and * p value < 0.05.


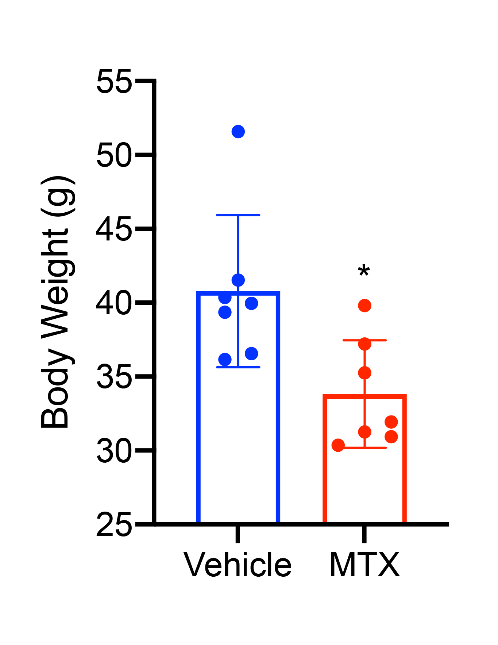


**Supplemental Figure 2.** **Body weight of mice on HFD treated once a week with vehicle or MTX for 20 weeks.**

Body weight of mice on HFD treated once a week with either vehicle or with 1mg/kg MTX for 20 weeks. Experiments were performed with C57BL/6J male mice, n=7 per group. Results are expressed as a mean ± SEM and * p value < 0.05; ** p value < 0.005.

**Supplemental Figure 3.** **Analysis of liver enzymes.**

Levels of ALT and AST enzymes present in serum obtained from vehicle- and MTX-treated mice on HFD. Experiments were performed with C57BL/6J male mice, n=7 per group.


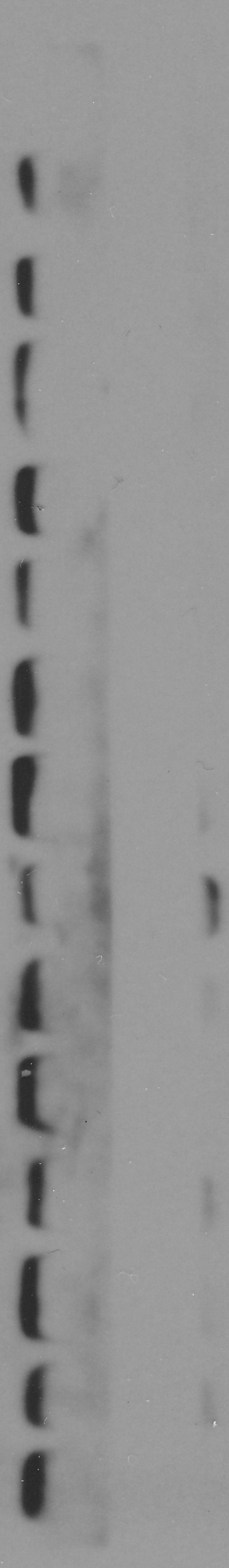

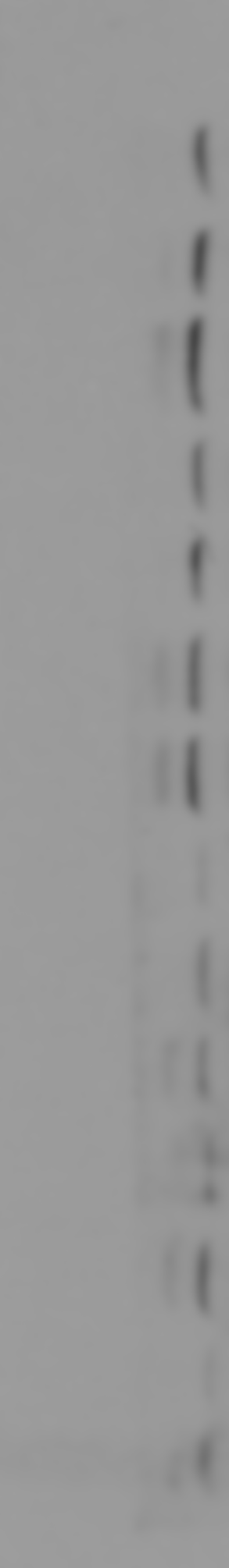


P-AKT (S473)

Vinculin

AKT


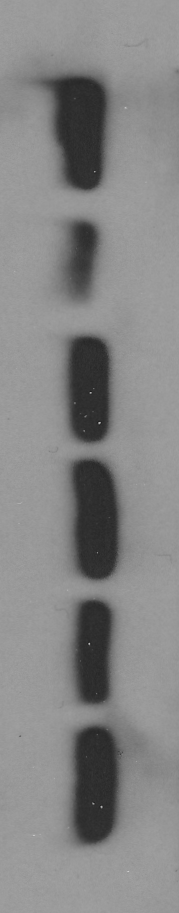


Liver

Vehicle

MTX


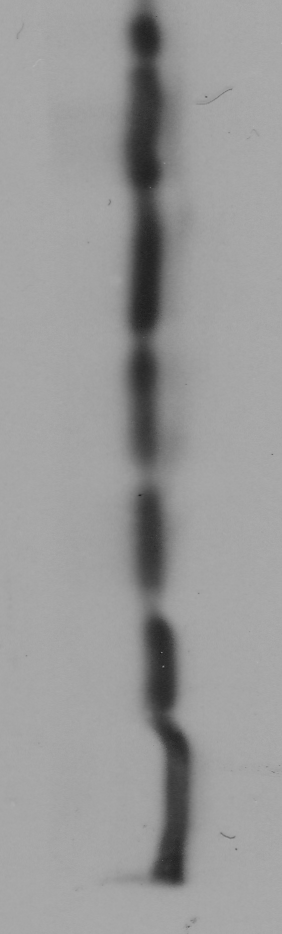


AMPK

Vinculin


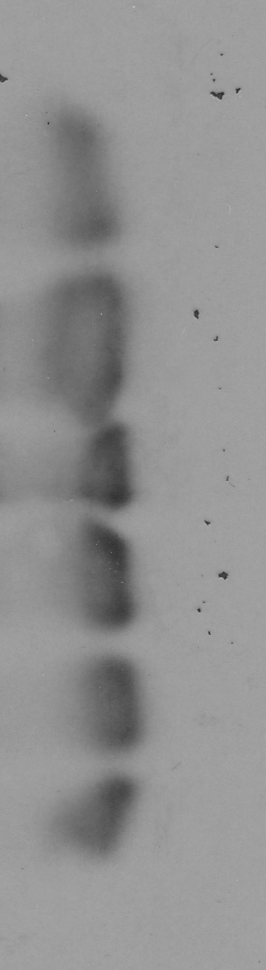


Vehicle

MTX

**A**

**B**

Muscle

**Supplemental Figure 4.** **Levels of** **AKT and phospho-AKT in livers and of AMPK in muscle of mice on HFD, treated once a week with vehicle or with MTX for 20 weeks.**

Western blot analysis of AKT and of phospho-AKT protein levels in liver (A) and of AMPK levels in muscle (B) and their quantification via densitometric analysis. Tissues analyzed were obtained from mice exposed to HFD for 20 weeks while treated with vehicle or MTX once a week. Vinculin was used as loading control. Results are expressed as a mean ± SEM. ** p value < 0.005.

**Supplemental Figure 5.** **Weights of tissues obtained from mice on HFD for 20 weeks, treated once a week with vehicle or MTX.**

(**A**) Spleen, muscle, pancreas, brain, heart tissue weights of mice on HFD treated with vehicle or 1mg/kg MTX for 20 weeks, once a week. Experiments were performed with C57BL/6J male mice, n=7 per group. Results are expressed as a mean ± SEM.

**Supplemental Figure 6. Analysis of markers of fibrosis in liver biopsies obtained from mice on HFD for 20 weeks, either treated with vehicle or MTX once a week.**

mRNA levels of Timp2 and MMP14 in liver tissues obtained from mice on HFD, either treated with vehicle or MTX. n=7 per group
